# Assessing the role of *REM13*, *REM34* and *REM46* during the transition to the reproductive phase in *Arabidopsis thaliana*

**DOI:** 10.1007/s11103-023-01357-1

**Published:** 2023-05-12

**Authors:** Silvia Manrique, Francesca Caselli, Luis Matías-Hernández, Robert G. Franks, Lucia Colombo, Veronica Gregis

**Affiliations:** 1 Dipartimento di Bioscienze, Università degli Studi di Milano, Via Giovanni Celoria 26, 20133 Milan, Italy; 2 Department of Plant and Microbial Biology, North Carolina State University, 27606 Raleigh, NC USA; 3 Tricopharming, C/Pallars 99, 08018 Barcelona, Spain

**Keywords:** *REM* transcription factor, Flowering time, Cell division, Co-expression analysis, Protein interaction, *Arabidopsis thaliana*

## Abstract

REM (reproductive meristem) transcription factors have been proposed as regulators of plant reproductive development mainly based on their specific expression patterns in reproductive structures, but their roles are still largely unknown probably because of their redundancy. We selected three *REM* genes (*REM13*, *REM34* and *REM46*) for functional analysis, based on their genome position and/or co-expression data.

Our results suggest that these genes have a role in flowering time regulation and may modulate cell cycle progression. In addition, protein interaction experiments revealed that *REM34* and *REM46* interact with each other, suggesting that they might work cooperatively to regulate cell division during inflorescence meristem commitment.

Previous attempts of using co-expression data as a guide for functional analysis of REMs were limited by the transcriptomic data available at the time. Our results uncover previously unknown functions of three members of the *REM* family of *Arabidopsis thaliana* and open the door to more comprehensive studies of the REM family, where the combination of co-expression analysis followed by functional studies might contribute to uncovering the biological roles of these proteins and the relationship among them.

**Supplementary Information:**

The online version contains supplementary material available at 10.1007/s11103-023-01357-1.

## Key message

*REM13, REM34* and *REM46* were selected, through co-expression analysis of the whole REM family, as involved in flowering time regulation and show alteration in the cell division rate during this process.

**Supplementary Information:**

The online version contains supplementary material available at 10.1007/s11103-023-01357-1.

## Introduction

REM (Reproductive Meristem) proteins belong to the plant-specific B3 superfamily of transcription factors (Franco-Zorrilla et al. 1999). *REM* genes appear consistently in transcriptomic studies focused on reproductive development (Mantegazza et al. 2014; Wynn et al. 2011), but their roles are still largely unknown. Currently, no phenotypes have been observed for several single or higher-order mutants, even if the *REM* genes under study were strongly expressed during specific phases and/or tissues during reproductive development (Mantegazza et al. 2014; Romanel et al. 2011; Wynn et al. 2011). Functional redundancy might contribute to this, as all investigated plant genomes contain dozens of *REM* genes often located in tandem in the genome, suggesting they arose by fragment duplications (Ahmad et al. 2019; Romanel et al. 2009; Ruan et al. 2021; Swaminathan et al. 2008; Verma and Bhatia 2019; Wang et al. 2012).

*REM* transcription factors are characterized by the presence of multiple B3 domains, a plant-specific DNA binding domain (Swaminathan et al. 2008), but differently to other families in the B3 superfamily, like ARFs, the mechanism for DNA binding of REM proteins is unclear, as different modes of binding have been described. While VRN1 was reported to bind DNA in a non-sequence specific mode (Levy et al. 2002) and, more recently, to undergo liquid-liquid phase separation with DNA (Zhou et al. 2019), REM1/REM34 binds DNA in a sequence-specific mode (Franco-Zorrilla et al. 2014). In addition, many of the REM proteins characterized so far can homo- and heterodimerize (Caselli et al. 2019; Mendes et al. 2016), suggesting that dimerization might be a common mechanism of action in this family.

So far, only a handful of *REM* genes have known roles in reproductive development. A few *REMs* have assigned roles in ovule development. Namely, in Arabidopsis, *VERDANDI* (*VDD*, *REM20*) (Matias-Hernandez et al. 2010), *VALKYRIE* (*VAL*, *REM11*) (Mendes et al. 2016), *REM34* and *REM35* (Caselli et al. 2019) have been linked to gametophyte development, whereas *REM22* contributes to ovule primordia formation (Gomez et al. 2018). In rice, *OsREM20* was recently shown to regulate grain number per panicle (Wu et al. 2021).

The other few REM genes functionally characterized include*VERNALIZATION1* (*VRN1*/*REM5*) (Levy 2002), *RELATED TO VERNALIZATION 1* (*RTV1*/*REM4*) (Heo et al. 2012), *TARGET OF FLC AND SVP 1* (*TFS1*/*REM17*) (Richter et al. 2019), and *REM16* (Yu et al. 2020), all involved in flowering time regulation.

Floral transition is considered one of the most important developmental switches undertaken by the shoot apical meristem (SAM) and it affects the number of leaves, flowers and fruits produced by a plant. After the vegetative to reproductive transition, the SAM remodels into an inflorescence meristem (IM) which has the ability to initiate floral meristems (FMs) that will give rise to flowers (Fornara et al. 2010; Kwiatkowska 2008). This transition has to be precisely coordinated through internal and external signals, to ensure the reproductive success of the plant. A broad range of studies done in *Arabidopsis thaliana* and expanded to crops and other species suggest substantial conservation of this process (Blümel et al. 2015). Several signals such as developmental, hormonal and environmental cues are precisely perceived and processed from six main genetic pathways, which converge into a few genes called “floral integrators” (Simpson and Dean 2002). Control of flowering in response to seasonal changes is mastered by the vernalization and photoperiod pathways, whereas changes in ambient temperature are perceived by the ambient temperature pathway. In *Arabidopsis thaliana*, flowering is promoted by long-day photoperiod (LD, when night length falls below a certain threshold), but this is not a mandatory requirement. Therefore, Arabidopsis plants grown in short-day photoperiod (SD) will eventually flower as well (Wang et al. 2019). The age, autonomous, and gibberellin pathways, act mainly independently of environmental stimuli (Srikanth and Schmid 2011). The synergetic action of these pathways ultimately converges into floral integrator genes which modulate flowering time and activate floral meristem identity genes (Srikanth and Schmid 2011). *FLOWERING LOCUS T* (*FT*), which belongs to the PEBP (phosphatidylethanolamine-binding protein) family (Kobayashi et al. 1999), and *SUPPRESSOR OF OVEREXPRESSION OF CONSTANS 1* (*SOC1*/*AGL20*) (Lee et al. 2000), a MADS-box transcription factor, are two floral integrators; FT interacts with FLOWERING LOCUS D (FD) in the SAM to activate the floral promoter SOC1 and, later on, the floral meristem identity genes *APETALA 1* (*AP1*) and the *AP1* paralog *CAULIFLOWER* (*CAL*) (Abe et al. 2005). SOC1 also activates floral meristem identity through *LEAFY* (*LFY*). Together, *LFY*, *AP1*, *CAL*, *AGL24* and *SVP* orchestrate the differentiation of a group of cells at the flank of IM into FMs (Gregis et al. 2008; Liu et al. 2008).

During the vegetative to reproductive transition, an increase in cell division occurs in the SAM (Jacqmard et al. 2003; Kwiatkowska 2008). This phenomenon was first described in the 1960s (Bernier 1969; Corson, 1969; Miksche and Brown, 1965) at the cytological level, and since then it has been described in a variety of plants suggesting that it is widespread among angiosperms (Kurokura et al. 2006; Lyndon and Battey 1985; Marc and Palmer, 1984). A more recent study in *Arabidopsis thaliana* (Jacqmard et al. 2003), showed that, upon transfer to LD, the increase of mitotic activity is one of the earliest events at the SAM in the vegetative to reproductive transition, preceding the enlargement and doming of the SAM and the bolting and initiation of the first floral meristem (Jacqmard et al. 2003). The authors found also that mitotic activity increases throughout the SAM, except in the organizing center, where stem cells reside (Jacqmard et al. 2003).

However, in all these pioneering works, molecular events were not characterized. In 2015, Klepikova et al. published a study based on RNA-seq, where they found that the increase in cell division observed during floral transition is supported by the upregulation of genes related to the cell cycle that occurs at a precise stage, where *FLC* expression has decreased and *LFY* expression has not started to increase yet. Based on the expression patterns of cell cycle-related genes, the authors proposed that the increase in cell division was caused by a shortening of G1 and G2 phases, which also causes a partial synchronization of the cell cycle. This hypothesis is also in line with most of the previous observations by cytological means. More recently, Kinoshita et al. (2020) showed that the increase in cell division is, at least, partially controlled by the photoperiodic and the gibberellin flowering pathways.

In this work, we selected three *REM* genes (*REM13*, *REM34* and *REM46*) for functional analysis based on their genome position or co-expression data. Our results indicate that these genes have a role in flowering time regulation and may modulate cell cycle progression. In addition, protein interaction experiments revealed that REM34 and REM46 interact with each other, suggesting that they might work cooperatively. Overall, our findings add pivotal information about the biological role of three members of the REM family of *Arabidopsis thaliana* and the co-expression patterns of the *REM* family, which might contribute to uncovering the biological roles of these genes and the functional relationship among them.

## Materials and methods

### Plant material and growth conditions

Mutant lines for the different *REMs* were obtained from the Nottingham Arabidopsis Stock Center (NASC): SALK_022885 (*rem13-1/rem13_oe*), SALK_050242 (*rem13-2/rem13_kd*), FLAG_566H04 (*rem34-1*), and SALK_151966 (*rem46-1*). Primers for the genotyping of mutants are listed in Supplementary Table 1. For flowering time assays plants were grown in a growth chamber under long-day conditions (LD: 16 h light/8 h darkness) at 22/24 °C. For expression analysis and cell cycle analysis, plants were grown in a growth chamber under short-day conditions (SD: 8 h light/16 h darkness) for 21 days. Then, plants were transferred to long-day conditions to induce flowering.

### Co-expression and GO term enrichment analysis

For co-expression analysis, the “co-expression neighborhood” (CEN, list of the 20 most closely co-expressed genes with a particular gene) of each *REM* gene was retrieved from the *athrna* database (Zhang et al. 2020) and the number of co-expressed genes shared between a particular *REM* gene with every other *REM* gene was used for the clustering analysis. Clustering analysis and heatmaps were obtained with ClustVis, using Euclidean distances and average linkage method. CENs for each *REM* gene can be found in Supplementary File 1 (downloaded on the 6th of September 2021 from *athrna* database). GO term enrichment analysis was performed using Panther (Mi et al. 2019a, b).

### Flowering time measures

Flowering time was measured in terms of the number of rosette leaves at the time of bolting and of days from sowing to bolting. Plants were considered bolted and rosette leaves were counted when the inflorescence was 1 cm long. A minimum of 10 plants per genotype and replicate were used.

### Gene expression analysis

RNA was extracted using the LiCl method (Verwoerd et al. 1989). For each sample, 500 ng of RNA were retro-transcribed using iScript kit (BioRad) following the manufacturer’s instructions. qRT-PCR assay was performed using iTaq Universal SYBR Green supermix (BioRad) in a Bio-Rad iCycler iQ Optical System (software version 3.0a). Three biological replicates, with three technical replicates for each sample, were analyzed. Relative transcript enrichment of genes of interest was calculated by normalizing the amount of mRNA against *EIF4* (Yamaguchi et al. 2009). Expression of genes was calculated using the 2^−ΔΔCt^ method, using the Wild Type or Wild Type T0 as normalizer. Statistical significance was calculated on ΔCt values with a t-test. The primers used for this analysis are listed in Supplementary Table 1.

### In situ hybridization analysis

SAMs were dissected by removing fully developed leaves, fixed in FAA (50% ethanol, 5% acetic acid, 3.7% formaldehyde) under vacuum for 15 min, dehydrated in ethanol and bioclear (Bioptica) and embedded in Paraplast Plus (Sigma-Aldrich). In situ hybridization was performed as previously described (Coen et al. 1990) with slight modifications. Digoxigenin-labelled antisense probes were synthesized with T7 RNA polymerase (Promega). For *REM13* detection we used the probe described in Villarino et al. 2016, for *REM34* we used the probe described in Mantegazza et al. 2014; for *REM46* we designed a new specific probe and for *H4* we employed the probe described in Petrella et al. 2020. Primers for probe amplification are listed in Supplementary Table 1. As the *REM46* probe was designed downstream of the *rem46-1* T-DNA insertion, specificity of the *REM46* probe was tested on the *rem46-1* mutant where, in contrast to wild type tissue, no signal was detected (Supplementary Fig. 5). The specificity of the signal given by all the other in situ hybridization probes employed in this study was already assessed in previous studies (Mantegazza et al. 2014; Robert et al. 1994; Villarino et al. 2016).

### Flow cytometry analysis of cell cycle

Plants were grown in a growth chamber under SD (non-inductive) conditions for 20 days. Then, the photoperiod was shifted to LD and samples were collected at three time points: before the light shift (T0), 24 h (T1) and 72 h (T3) after the transfer to inductive conditions. Each sample consisted of 3 meristems, with three technical replicates per time point and genotype. Samples for flow cytometry were prepared as described by Yang et al. 2019. Flow cytometry was performed using a BD FACS Canto II (BD Biosciences) equipped with FACSDiva Software v6.1.3. 10,000 events per sample were analyzed. Stages of the cell cycle in the nuclei population were determined using FlowJo® 10.8.1 (BD Life Sciences) using the Watson model.

### Plasmid construction

The coding sequences of *REM13, REM17, REM34, REM36* and *REM46* were amplified by PCR from cDNA, cloned in pDONR207 and subsequently transferred to pGADT7 and pGBKT7 (Clontech Laboratories, Inc) for yeast-two-hybrid assays; and to pYFN43 and pYFC43 (Belda-Palazón et al. 2012) for BiFC assays, by Gateway cloning (Invitrogen). Primers used for cloning are listed in Supplementary Table 1.

### Yeast-two-hybrid assay

The yeast-two-hybrid experiments were performed in the AH109 strain. The bait (pGBKT7, Clontech Laboratories, Inc) and prey (pGADT7, Clontech Laboratories, Inc) vectors were co-transformed in yeast as described by de Folter and Immink (de Folter and Immink 2011). The protein-protein interaction assays were performed on selective yeast synthetic dropout medium lacking leucine, tryptophan and histidine supplemented with different concentrations of 3-aminotriazole (1, 2.5, or 5 mM of 3‐AT). Plates were grown for 5 days at 28 °C. The already published REM34-REM34, REM34-REM35 and REM35-REM35 interactions were employed as negative and positive controls (Supplementary Fig. 4 and Caselli et al. 2019).

### Bimolecular Fluorescence Complementation (BiFC) assays

*Nicotiana benthamiana* leaves were infiltrated with *Agrobacterium tumefaciens GV3103* previously transformed with the vectors of interest and the viral suppressor p19. Three days after inoculation, the abaxial surface of the leaves was imaged employing a Laser Scanning Confocal Microscope Nikon A1. As for the yeast-two hybrid experiment, the already published REM34-REM34 and REM35-REM35 interactions were employed as negative and positive controls, respectively (Supplementary Fig. 4 and Caselli et al. 2019).

## Results

### REM13 is a positive regulator of floral transition

*REM13* (*AT3G46770*) is located on the long arm of chromosome 3. It is one of the few family members that is not part of a cluster of *REM* genes (Mantegazza et al. 2014) (Supplementary Fig. 1b), making it a good candidate for genetic analysis, as it could be potentially less redundant than other *REMs* that have undergone tandem duplications.

**Fig. 1:**
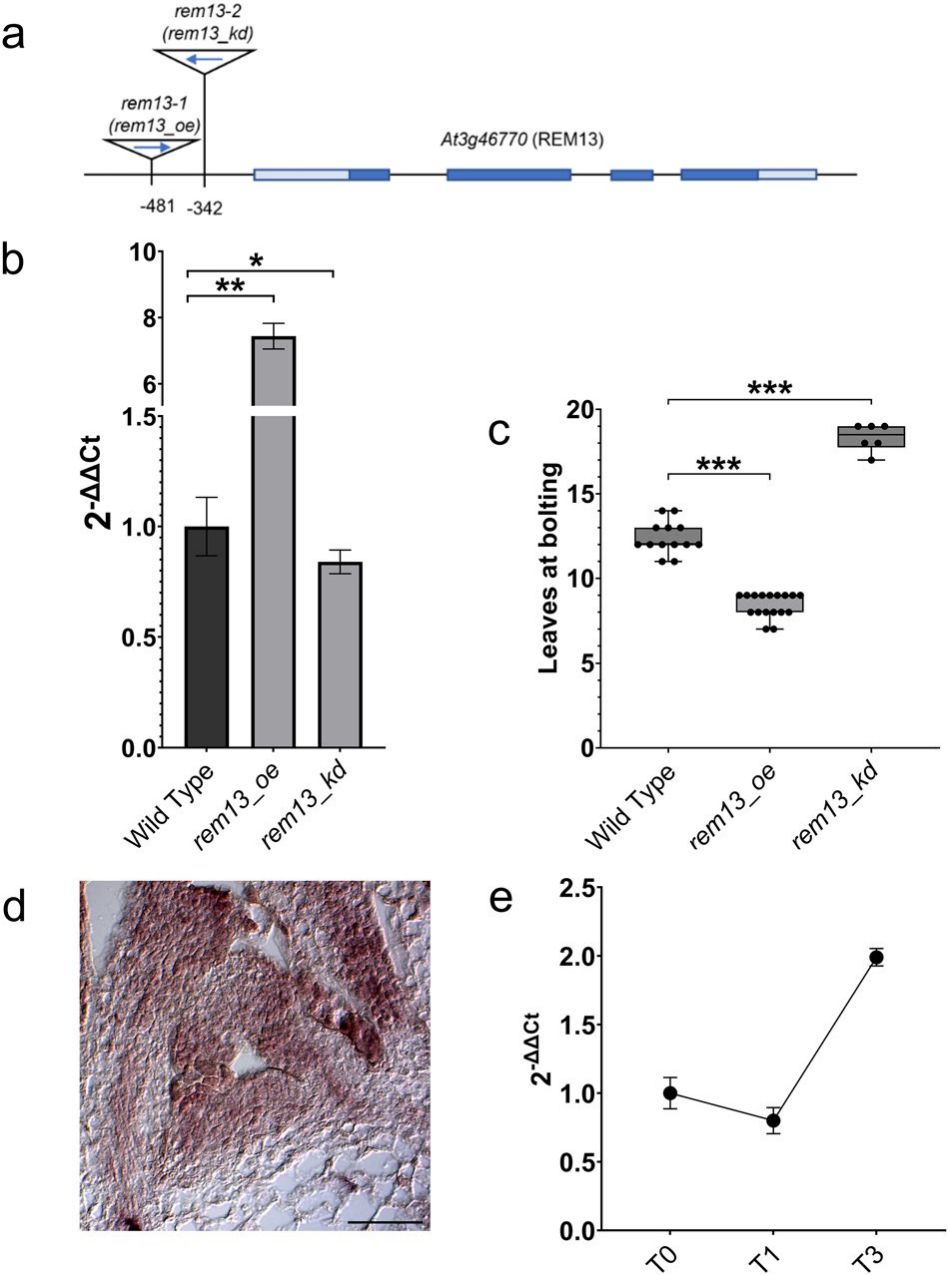
Characterization of *REM13* mutants. **a** Position of *rem13-1* (*rem13_oe*) and *rem13-2* (*rem13_kd*) T-DNA insertions in *REM13* (At3g46770) genomic region, light blue boxes: UTR, dark blue boxes: exons, lines: introns. **b** Expression of *REM13* in *rem13_oe* and *rem13_kd* mutants. Graph shows 2^−ΔΔCt^ average of three biological replicates, significance was calculated on ΔCt using t-test (* p-value < 0.05, ** < 0.01) **c** Flowering time of *rem13_oe* and *rem13_kd* mutants, measured as the number of rosette leaves at bolting. Significance was calculated using a t-test coupled with Bonferroni correction for multiple testing (*** p-value < 0.001). **d** *REM13* expression profile, detected by in situ hybridization in wild type SAM, showing *REM13* ubiquitously expression in the meristem and the adaxial side of developing leaves primordia. Scale bar 100 μm **e** Expression level of *REM13* in wild type SAM-enriched tissue in non-inductive SD conditions (T0) and 24 h (T1), 72 h (T3) after the switch to LD conditions. Graph shows 2^−ΔΔCt^ average of three biological replicates, normalized on T0

Unfortunately, lines with insertions or mutations in exons were not available in public collections. We thus analyzed two independent lines: SALK_022885 (*rem13-1*) and SALK_050242 (*rem13-2*), which contain insertions on the promoter region of *REM13*. The insertions were confirmed by sequencing to be at positions − 481 and − 342 respectively (Fig. 1a). The expression of *REM13* in these lines was analyzed by qRT-PCR and, interestingly, we found that, while *rem13-1* overexpressed *REM13* (henceforth called *rem13_oe* for “overexpressor”), *rem13-2* showed a moderate reduction (20%) of *REM13* expression level (henceforth called *rem13_kd* for “knock-down”) (Fig. 1b).

While growing the plants for propagation, we readily noticed that *rem13_oe* seemed to flower earlier than wild type plants. Therefore, we decided to analyze the flowering time of both mutant lines in inductive LD conditions (16 h light, 8 h dark) to verify the involvement of *REM13* in this character. While wild type plants produced on average 12.44 leaves at bolting, *rem13_oe* plants showed a reduction of flowering time, with plants bolting with an average of 8.39 leaves (Fig. 1c). Despite the moderate reduction in the expression of *REM13* displayed by *rem13_kd*, this line showed a delay in flowering time, with plants bolting with an average of 18.25 leaves (Fig. 1c). To better contextualize the putative role of *REM13* during the floral transition, the expression profile of this gene was investigated. In situ hybridization, carried out on wild type SAM, highlighted that *REM13* is expressed both in the meristematic dome and in the developing leaf primordia (Fig. 1d). Furthermore, we performed a time course qRT-PCR experiment, prepared from hand-dissected apices (SAM-enriched tissue) of wild type plants grown for three weeks in non-inductive SD conditions and transferred to LD to induce flowering. Samples were collected at T0 (SD conditions), T1 (24 h LD) and T3 (72 h LD), to assess the changes in the expression of the gene of interest during floral transition. This analysis showed that *REM13* mRNA level is stable during the switch between SD and LD (T0 and T1), and quickly rises 72 h after exposure to inductive conditions, further suggesting a putative role of *REM13* in the control of vegetative to reproductive transition (Fig. 1e). Overall, these results suggest that *REM13* promotes floral transition, since an increase in *REM13* expression leads to a reduction of flowering time, while its downregulation delays floral transition.

### Clustering analysis of REM genes supports independent roles in flowering time control

To better understand the redundancy relationship between the several *REMs* of Arabidopsis, and the role of *REM13* in the context of the whole *REM* family, we performed a co-expression analysis. First, we obtained a comprehensive list of all the *REM* genes that have been predicted in *Arabidopsis thaliana* (Romanel et al. 2009; Wang et al. 2012; Swaminathan et al. 2008). We checked the overlap between the lists and found that 44 *REM* genes were predicted by all the works, 10 by 2 works, and 22 were only predicted by Swamithanan and collaborators (Supplementary Fig. 1a). One of the predicted genes (AT2G21920) (Swaminathan et al. 2008) is presently annotated as an F-box protein, so we removed it from the list. We generated a non-redundant list containing the 75 predicted *REM* genes (Supplementary Table 2) and depicted their position in the chromosomes of *A. thaliana* (Supplementary Fig. 1b). Romanel and collaborators named the 45 *REM* genes they described as *REM1-45* (Romanel et al. 2009). *REM46* was named in a separate work (Villarino et al. 2016), while the remaining 29 *REM* genes were not named, so we kept this nomenclature in this work.

**Fig. 2:**
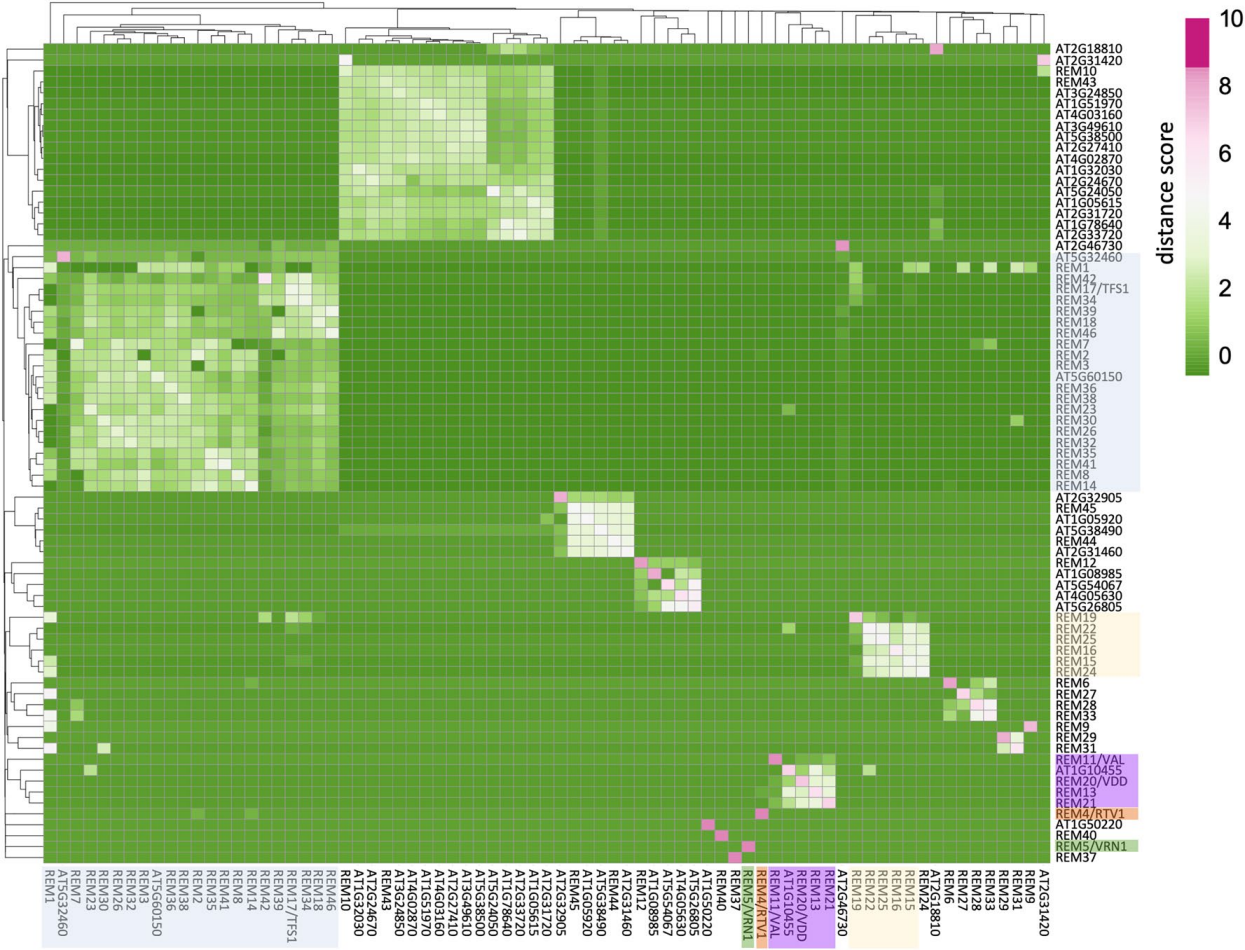
Co-expression matrix for *REM* genes. Co-expression data was extracted from *athrna* database. Columns and rows were hierarchically clustered using Euclidean distance and the average linkage method using ClustVis (biit.cs.ut.ee/clustvis/) (Metsalu and Vilo 2015). *REM13* cluster is highlighted in purple. *REM17/TFS1* (light blue), *REM16* (pink), *REM4/RTV1* (orange) and *REM5/VRN1* (green) and their respective clusters are also highlighted, as they have also been linked with flowering time regulation

Recently, Zhang and collaborators published a database (*athrna* database) based on more than 20,000 publicly available RNA-seq (Zhang et al. 2020). *athrna* database can generate a list of the 20-top co-expressed genes with a particular gene (from here on its “co-expression neighborhood”, “CEN”). Therefore, to gain further insight into the function of *REM13*, we performed a co-expression analysis based on the CENs provided by *athrna* database (Supplementary Table 3) and we produced a distance matrix comparing 75 *REM* genes using ClustVis (Metsalu and Vilo 2015) (Fig. 2). This analysis revealed that several groups of *REMs* cluster together, as their CENs share numerous genes.

*REM13* forms a small cluster (highlighted in purple in Fig. 2) with *REM11/VAL*, *REM20/VDD*, *REM21* and *AT1G10455*. None of these genes have been linked to flowering time regulation. *REM20/VDD* and *REM11/VAL* modulate synergid degeneration upon pollen tube arrival (Matias-Hernandez et al. 2010; Mendes et al. 2016), while *REM21* and *AT1G10455* do not have a known function yet.

We also investigated the *REM* genes already known to be involved in flowering time. *TFS1/REM17*, an activator of floral transition and target of FLC and SVP (Richter et al. 2019), is located in a big cluster containing 22 *REMs* in total (highlighted in light blue in Fig. 2). In this cluster, besides *TFS1*, the only genes with known biological roles are *REM34*, *REM35*, and *REM36* which have been related to gametophyte development (Caselli et al. 2019).

*REM16*, a promoter of flowering that regulates *SOC1* and *FT* (Yu et al. 2020), forms a small group of 6 genes with *REM15, REM19*, *REM22*, *REM24* and *REM25* (highlighted in pink in Fig. 2). Among these, *REM15* was found to be a target of AGAMOUS (Gómez-Mena et al. 2005) expressed in the megaspore mother cell (Wynn et al. 2011). Instead, *REM22* and *REM24* were upregulated in the gibberellin-insensitive mutant *gai-1*, which is involved in the regulation of ovule number (Gomez et al. 2018).

Finally, *REM5/VRN1* and *REM4/RTV1*, which regulate flowering time through the vernalization pathway (Heo et al. 2012; Levy et al. 2002), do not cluster among them or with any other *REMs*.

In summary, co-expression analysis based on *athrna* data showed that *REM13* does not cluster with any of the *REM* genes previously linked with floral transition, suggesting that it might regulate flowering time through an independent pathway. In addition, some of the genes that cluster with *REM13*, like *VDD/REM20* or *VAL/REM11*, are involved in other processes along the reproductive phase. Interestingly, this same pattern was observed for the rest of the *REMs* with a known role in floral transition (*TFS1/REM17*, *REM16*, *VRN1* and *RTV1*), as all of them clustered separately and the respective clusters contained *REMs* with functions in other reproductive phases. Overall, this observation suggests two conclusions. First, that all the *REMs* with a known role in floral transition act independently of each other, and second, that either REMs have recursive roles along the reproductive process, or that different *REMs* might regulate different processes through the control of similar sets of genes.

### REM34 and REM46 are negative regulators of floral transition

The co-expression analysis revealed that *TFS1/REM17*, known to be involved in flowering time regulation (Richter el al., 2019), belongs to a cluster containing 22 *REM* genes (Fig. 2). Interestingly, some of the genes of the cluster are linked to other processes throughout Arabidopsis life cycle, like *REM34* (Caselli et al. 2019), involved in gametophyte development, or *REM46*, which was found to be a marker gene for the carpel margin meristem (Villarino et al. 2016). This poses the question of whether each of these *REM* genes regulates more than one process along reproduction, or if each one acts in a specific phase, but they all exert their function through the control of similar sets of genes. To investigate this, we examined whether *REM34* and *REM46* also have a role in flowering time control.

**Fig. 3:**
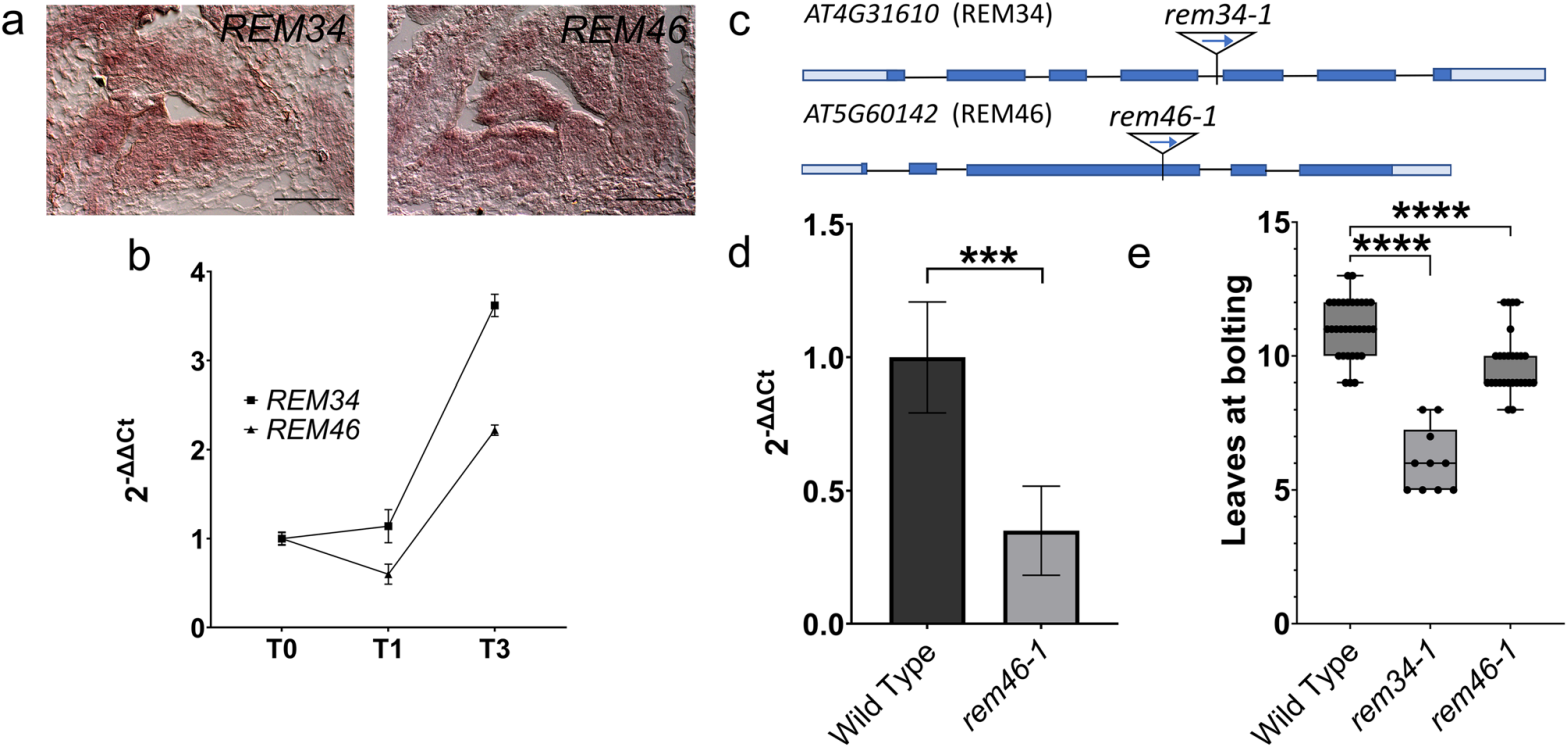
Characterization of *rem34-1* and *rem46-1*. **a** in situ hybridization analysis showing the expression profiles of *REM34* and *REM46* within the SAM and the adaxial side of developing leaves primordia for *REM34* and the whole developing leaves primordia for *REM46*. Scale bar: 100 μm (Control for the *REM46* probe is presented in Supplementary Fig. 5) **b** RT-PCR showing the expression level of *REM34* and *REM46* in wild type SAM-enriched tissue in non-inductive SD conditions (T0) and 24 h (T1), 72 h (T3) after the switch to LD conditions. Graph shows 2^−ΔΔCt^ average of three biological replicates, normalized on T0. **c** Position of *rem34-1* and *rem46-1* T-DNA insertion in the *REM34* (AT4G31610) and *REM46* (AT5G60142) genomic sequences. Light boxes: UTR, dark boxes: exons, line: introns. **d** Expression of *REM46* in *rem46-1*, compared to the wild type, *rem46-1* shows a reduction in the expression of around 70%. significance was calculated on ΔCt using t-test (***p-value < 0.001) **d** Flowering time of wild type, *rem34-1* and *rem46-1* plants measured as leaves at bolting. Significance was calculated using a t-test coupled with Bonferroni correction for multiple testing. (****p-value < 0.0001)

First, we verified the expression pattern of these two genes in the SAM during floral transition. In situ hybridization revealed that *REM34* and *REM46* share a similar expression domain, being expressed mainly in the meristematic dome (Fig. 3a, control in supplementary Fig. 5). A qRT-PCR time-course analysis revealed that *REM34* and *REM46* mRNA level is steady during the shift between SD (T0) and LD (24 h LD) and it increases in T3 (72 h LD) (Fig. 3b).

We then analyzed whether flowering time was altered in *rem34-1* and *rem46-1* mutant lines. For *REM34*, we employed the already characterized FLAG_566H04 line (*rem34-1)*, which shows downregulation of the *REM34* transcript (Mantegazza et al. 2014). For *REM46*, we chose the uncharacterized SALK_151966 line (*rem46-1* from here on), which carries a T-DNA insertion in an exonic region in the four splice forms of *REM46* (Fig. 3c). The insertion is located at the end of the third exon in the canonical splice isoform (AT5G60142.1) and causes a 70% reduction of *REM46* transcript level (Fig. 3d).

We analyzed flowering time under LD conditions in *rem34-1* and *rem46-1* and we observed that both mutants showed an early flowering phenotype compared to wild type plants which, in our growing conditions, had on average 11.06 leaves at bolting. r*em46-1* produced indeed an average of 9.82 leaves at the bolting stage, while *rem34-1* showed a higher reduction, having 6.1 leaves at bolting (Fig. 3e). The negative effect on flowering time regulation by *REM34* was confirmed by analyzing three *REM_RNAi* lines and two *35S:REM34-EAR* fusion lines, previously described by Caselli et al. 2019 (Supplementary Fig. 2). In conclusion, our results suggest that *REM34* and *REM46* share a similar expression profile throughout the floral transition, and they act as negative regulators of this process.

### Expression of floral transition markers in the early flowering REM mutants

To better understand the role of *REM13, REM34* and *REM46* during the reproductive switch, we measured the expression of three floral integrators in the *rem* mutant backgrounds of interest. To synchronize the plants in order to compare the different genotypes, the plants were grown under non-inductive SD conditions for 21 days and then switched to LD conditions to induce flowering. As described above, SAM-enriched tissue was collected at 0 h (T0), 24 h (T1) and 72 h (T3) after the transfer from SD to LD conditions.

**Fig. 4:**
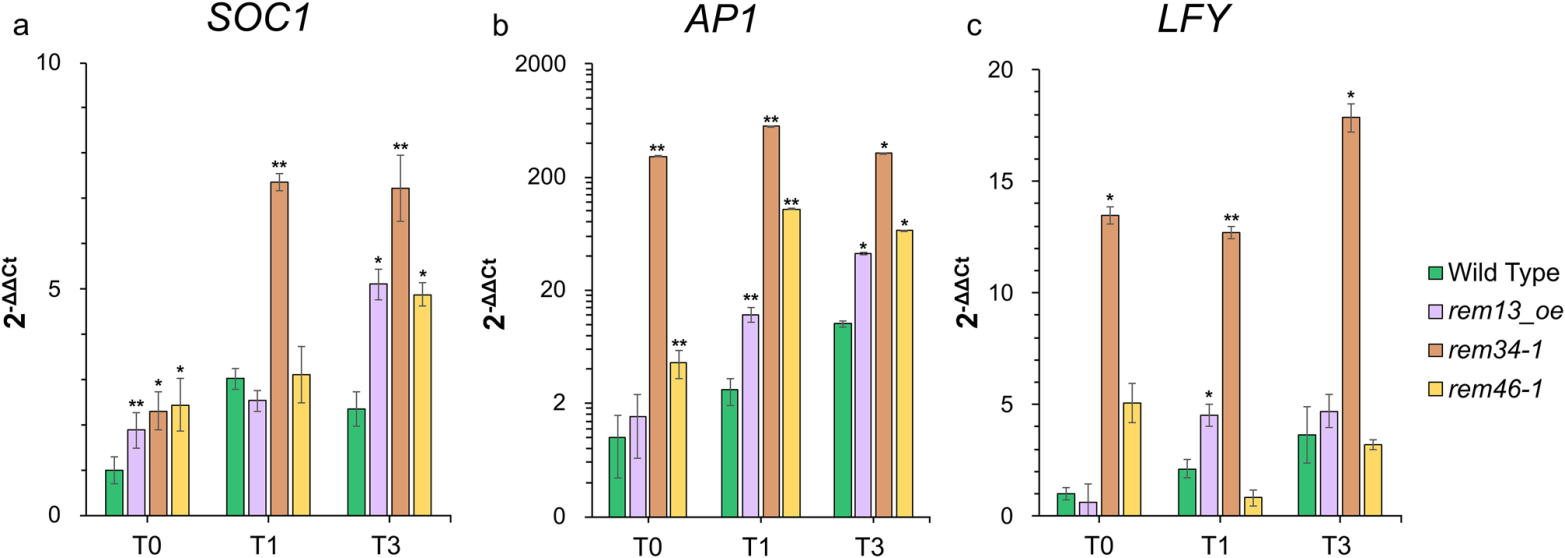
Expression analysis of *SOC1* (**a**), *AP1* (**b**) and *LFY* (**c**) in the wild type, *rem13_oe, rem34-1* and *rem46-1* genetic backgrounds. The expression levels are normalized on the *EIF4* housekeeping gene and wild type at T0 was set to 1. Graph shows 2^−ΔΔCt^ average of three biological replicates, significance was calculated on ΔCt using t-test (* p-value < 0.05, ** < 0.01, *** < 0.001)

The MADS-box gene *SOC1* marks the floral transition, as it starts to be expressed at T1, as soon as the SAM acquires the competence to become IM (Yoo et al. 2005). In the early flowering mutants *rem34-1* and *rem46-1*, as well as in *rem13_oe*, *SOC1* expression at T0, before the photoperiodic induction, is higher than in the wild type. *SOC1* upregulation was particularly dramatic in all of the analyzed time points in the *rem34-1* background, which is the mutant showing the earliest flowering phenotype (Fig. 4a).

As a second marker, we used the floral meristem identity gene *AP1* (Liljegren et al. 1999). In wild type SAMs, *AP1* expression starts to increase at T1 and reaches its highest level of expression 72 h after the switch to LD (T3). The *AP1* transcript was highly upregulated already at T0 in *rem34-1* and is continuously upregulated in the subsequent time points. *AP1* was slightly upregulated also in *rem46-1* in all three time points. In *rem13_oe*, *AP1* transcript is upregulated at T1 and T3 compared to the wild type, suggesting that also in this line the floral meristem starts to be specified earlier (Fig. 4b).

Finally, we analyzed the expression of *LFY* (Liljegren et al. 1999). *LFY* was upregulated in *rem34-1* throughout all the time points analyzed while in *rem13_oe* a slight upregulation was visible at T1. *rem46-1* instead showed a pattern similar to the wild type (Fig. 4c).

These data fit with the phenotypical analysis, as in the early flowering mutants *rem34-1*, *rem46-1*, and *rem13_oe*, most or all the analyzed floral transition markers show higher and/or earlier expression than the wild type.

Moreover, in *rem34-1*, which has the most drastic reduction of the flowering time, *SOC1, LFY* and *AP1* were strongly upregulated already at T0, before the switch to LD conditions. To verify whether this upregulation correlates with an early flowering time in non-inductive SD conditions, wild type and *rem34-1* plants were grown in SD and bolting time was recorded. *rem34-1* plants started to bolt 45 days after germination, while wild type plants remained in a vegetative state (Supplementary Fig. 3a).

### Co-expression neighborhoods link REM clusters to specific biological processes

**Fig. 5:**
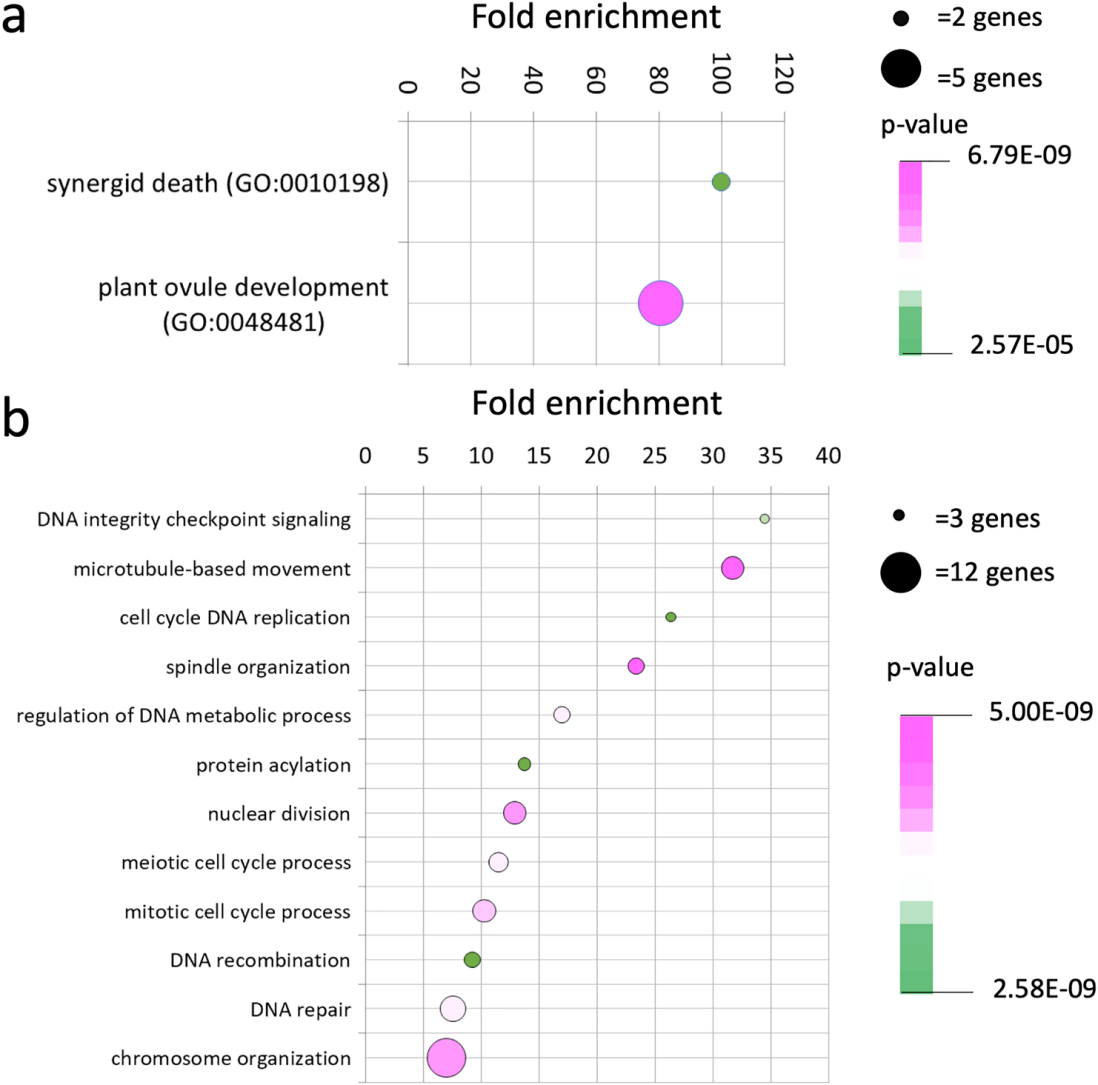
GO term enrichment analysis of the 20-top co-expressed genes (“CEN”) with *REM13* (**a**) and *REM17/TFS1* (**b**) clusters. Enrichment analysis was performed using Panther (Mi et al. 2018). For simplicity, only tip-most GO terms are plotted

To gain further insight into how *REM13*, *REM34* and *REM46* regulate flowering time, we performed an enrichment analysis of Biological Process GO terms associated with the genes belonging to the CENs of the clusters containing *REM13*, *REM34* and *REM46*. For that, we obtained non-redundant lists of all the genes in the CENs of every *REM* belonging to the clusters of interest and performed an enrichment analysis using Panther (Mi et al. 2019a, b) (Fig. 5, Supplementary Table 4).

In the case of the *REM13* cluster (Fig. 5a), the terms ‘synergid cell death’ and ‘plant ovule development’ were highly enriched, in accordance with the presence of *VDD* and *VAL*, but no terms that can be linked to flowering time were found.

*REM34* and *REM46* belong to the same cluster (Fig. 2). *REM* genes in this cluster are co-expressed with genes associated with cell cycle progression (Fig. 5b), as GO terms like ‘DNA integrity checkpoint signaling’, ‘cell cycle replication’, or ‘mitotic cell cycle process’ were enriched. Terms like ‘cell cycle DNA replication’, ‘microtubule-based movement’, ‘spindle organization’ or ‘nuclear division’ seem to suggest a role in the replication (S phase) and division (M phase) phases of the cycle. Moreover, these genes might be also involved in meiosis, as terms linked to meiosis like ‘DNA recombination’ or ‘meiotic cell cycle process’ are also enriched. Although no terms explicitly linked to flowering time control were found, the floral transition involves an increase in cell division at the SAM (Jacqmard et al. 2003; Kinoshita et al. 2020; Klepikova et al. 2015; Kwiatkowska 2008), suggesting that the role of *REM* genes located in this cluster might be linked to cell division.

### REM13, REM34 and REM46 modulate cell cycle progression in the SAM during floral transition

During the transition from the vegetative SAM to the IM, there is an increase in cell division (Kinoshita et al. 2020; Klepikova et al. 2015; Marc and Palmer, 1984). GO term enrichment analysis suggested that *REM34* and *REM46* might be involved in the regulation of the cell cycle (Fig. 5b), which could be linked to the early flowering time phenotype of these mutants.

**Fig. 6:**
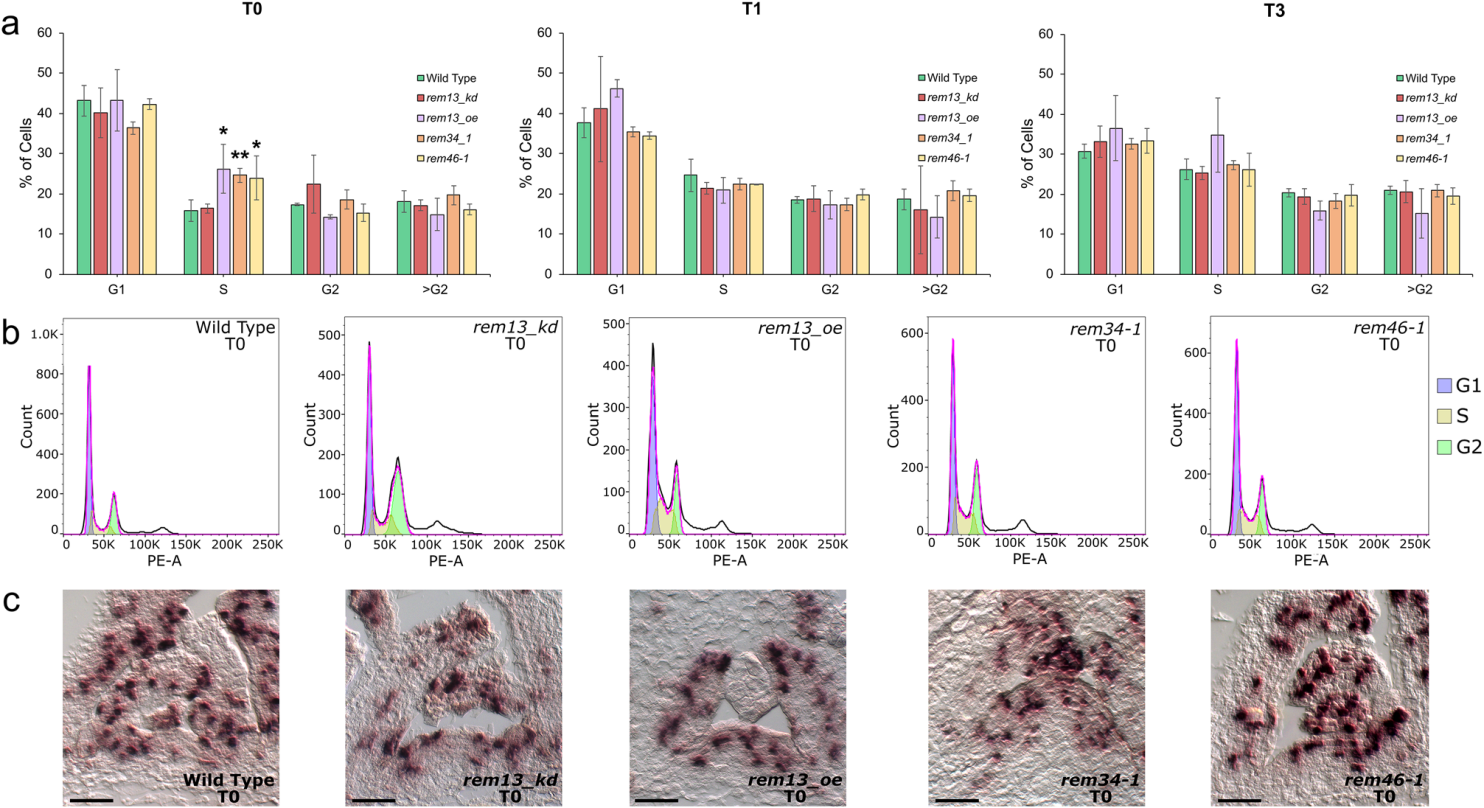
Cell cycle progression analysis. **a** The histograms show the percentage of cells in the different cell cycle stages (G1, S, G2, >G2) at T0, T1 and T3 as the average of three biological replicates, each one consisting of three meristems. At T0, *rem13_oe, rem34-1* and *rem46-1* exhibit a significative increase in the percentage of cells in the S phase, consistent with an increase of cell division rate. Statistical significance was evaluated with a t-test (*p < 0.05, **<0.01). **b** shows an example of the nuclei distribution and their difference in DNA content at T0 for each genotype. **c** Expression of the S phase marker *H4* was investigated via in situ hybridization, in meristems collected at T0 (SD conditions). In the wild type and *rem13_kd H4* show a similar expression pattern, as the cells expressing this marker are mainly localized in the peripheral zone of the meristem where the new leaf primordia are differentiating. In *rem13_oe, rem34-1* and *rem46-1*, however, the expression profile of H*4* is broader and cells expressing this marker are localized also in the central zone of the meristem, confirming a general increase in cell cycle rate. Scale bar 100 μm

To check if *REM13*, *REM34* and *REM46* can influence the cell cycle, we measured cell cycle progression in the meristem of wild type and mutants during the floral transition, through the estimation of DNA content by flow cytometry staining nuclei with propidium iodide (PI) (Fig. 6). In particular, we measured the cell cycle in hand-dissected apices before transferring the plants from SD to LD (T0), and 24 (T1) and 72 (T3) hours after the switch. The results were analyzed using FlowJo 10.8.1 (BD Life Sciences).

The wild type presented an increase in the percentage of cells in the S phase 24 h after the transfer from SD (T0) to LD conditions (T1) (Fig. 6a), which is symptomatic of an increased cell cycle rate associated with floral transition and is consistent with the increase in *SOC1* expression observed previously (Fig. 4a). The higher cell division rate is maintained also 72 h (T3) after transfer to LD (Fig. 6a) and it indicates that the SAM is enlarging and changing its identity, becoming the reproductive IM.

At T0, *rem34-1* and *rem46-1* showed already a 10% increase in the number of cells in the S phase compared with the wild type, suggesting that in the SAM of these mutants, cells divide faster than those of the wild type (Fig. 6ab). As the plants are shifted to LD (T1-T3), the cell cycling rate increases also in the wild type and the percentage of cells in the S phase reaches a similar level to *rem34-1* and *rem46-1*.

The two *rem13* mutant alleles, *rem13_kd* and *rem13_oe*, exhibit opposite behavior, in accordance with their different effects on flowering time regulation. In *rem13_oe*, indeed, which is characterized by a shorter flowering time, we measured a higher percentage of cells in S phase than in the wild type at T0, suggesting a possible increase in cell cycle also in this mutant. On the other hand, *rem13_kd* plants, characterized by a delayed flowering time, showed a similar trend of cell cycling and division of the wild type (Fig. 5 ab).

Overall, these results suggest that *REM13*, *REM34* and *REM46* are involved in the control of flowering time and show alterations in the progression of the cell cycle during floral transition. Furthermore, while *REM34* and *REM46* appear to act as negative regulators of cell cycle and division rate, *REM13* seems to promote these processes.

To further confirm the GO term enrichment and the flow cytometer analyses, which strongly suggested a link between the role of the *REM* genes under analysis in flowering time regulation and the modulation of cell division, the expression pattern of the *Histone4* (*H4*) mRNA was investigated by in situ hybridization (Fig. 6c). The *H4* is indeed considered a marker of cell cycle activity, being expressed specifically during the S phase (Geier et al. 2008; Wang and Liu 2006; Xu et al. 2008). The analysis was performed on tissues collected at T0, grown under short-day conditions, to allow the comparison of the results with the flow cytometer data. In the wild type SAM, the majority of the *H4*-expressing cells are located in the developing primordia, on the flanks of the meristematic area, where the cells are dividing and differentiating into leaves. In *rem13_kd* the *H4* expression pattern is similar to the one of the wild type, confirming that the number of cells in the S phase is similar in these two genetic backgrounds as suggested by the flow cytometer analysis. In *rem13_oe, rem34-1* and *rem46-1*, where a significative increase in the percentage of cells in the S phase was recorded, the expression profile of *H4* is wider than what was observed in the wild type. In particular, the cells expressing this cycling marker are not confined in the peripheral zone of the meristem but are spread into all three layers of the central zone, suggesting that the increase in the cell cycle rate observed in *rem13_oe, rem34-1* and *rem46-1* is due to an increase of dividing cells in the meristematic tissue. Furthermore, this analysis revealed a dramatic increase in the dimensions of the SAM of *rem34-1* compared to the wild type. The meristem, in this genetic background, appears to be both wider and higher and has a more pronounced dome than the one of the wild type, a shape that is usually observed in meristems that already underwent floral transition (Supplementary Fig. 3 bc). As the meristems employed for this analysis were collected under SD non-inductive conditions, this observation, coupled with the early flowering time phenotype observed in SD for *rem34-1*, strongly suggests that *REM34* might have a role in the light-dependent flowering time regulation.

### Interaction analysis of REM13, REM17, REM34 and REM46.

Several REMs are able to homo/heterodimerize (Caselli et al. 2019; Mendes et al. 2016). Therefore, we decided to test whether REM13, REM34 and REM46 can interact by yeast-two-hybrid (Y2H). Caselli and co-workers (Caselli et al. 2019) showed that REM34 heterodimerizes with REM35 but is unable to homodimerize, so these pairs were used as positive and negative controls respectively (Supplementary Fig. 4ab).

**Fig. 7:**
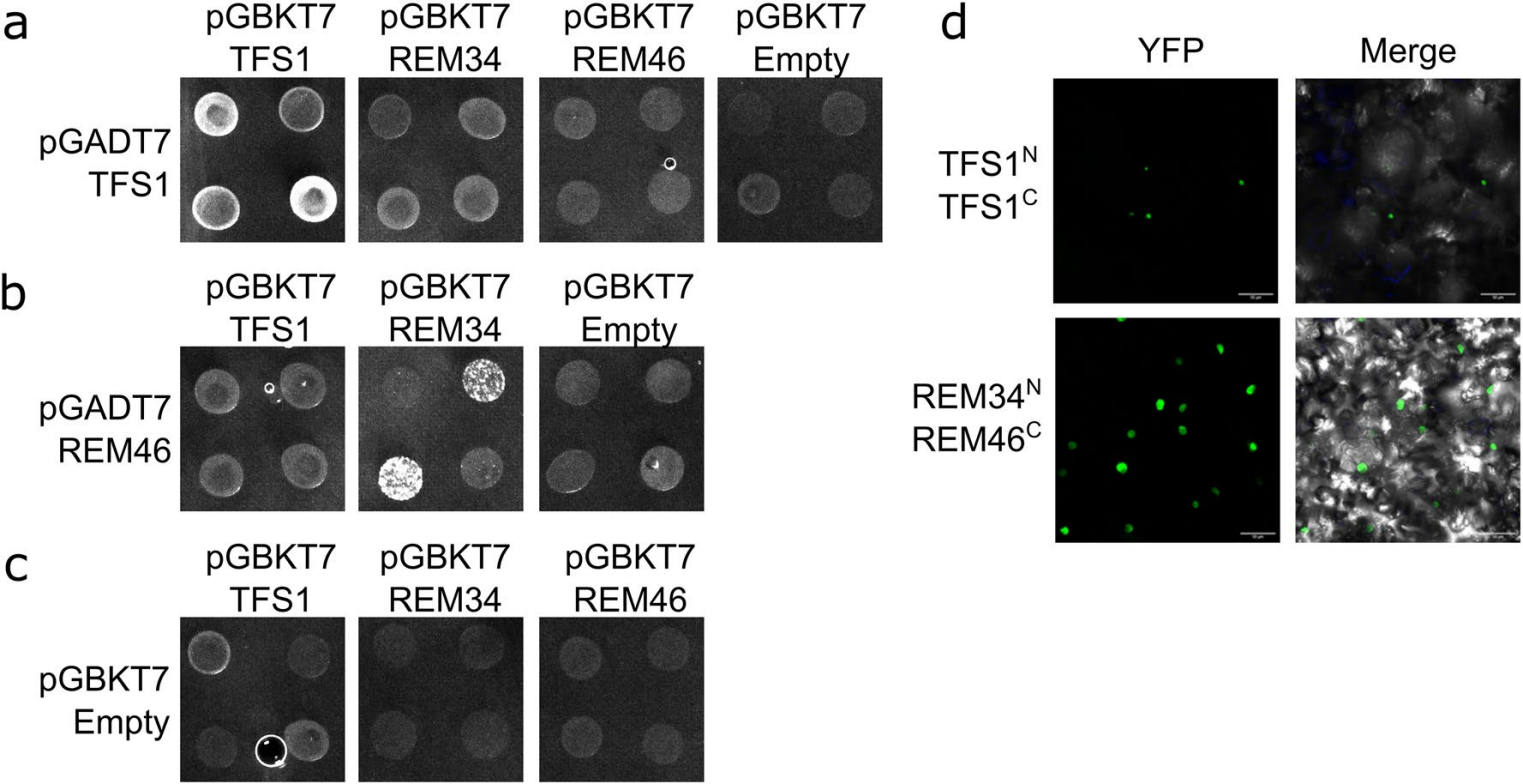
Protein-protein interactions. **a** Yeast-two-Hybrid assay showing that TFS1 is able to homodimerize but does not interact with REM34 and REM46, **b** REM46 can interact with REM34, **c** the empty pGADT7 and PGBKT7 were used as controls. For each tested interaction four independent colonies were tested on selective media lacking leucine, tryptophan and histidine, supplemented with different concentrations of 3-aminotriazole (1, 2.5, or 5 mM of 3‐AT) **d** BiCF assay, confirming the TFS1-TFS1 homodimerization and the REM34-REM46 heterodimers formation Bars = 50 µM

Y2H assays showed that REM46 can form heterodimers with REM34, suggesting that these proteins might cooperate (Fig. 7b). TFS1 was able to homodimerize but did not show any positive interaction with REM34 and REM46, even though they belong to the same co-expression cluster (Fig. 7a). This observation was in accordance with their opposite role in the regulation of flowering time (Richter et al. 2019).

Finally, in line with the co-expression data (Fig. 2), no interactions were found between REM13 and any of the other REMs tested (Supplementary Fig. 4b).

All the positive interactions found in the yeast-two hybrid analysis were further confirmed by Bimolecular Fluorescence Complementation (BiFC) (Fig. 7d). REM34-REM34 and REM34-REM35 interaction were used as negative and positive control respectively (Supplementary Fig. 4c).

The interaction between REM34 and REM46 indicates that these proteins might cooperate, as also suggested by the similar negative effect that these genes have on floral transition. Instead, in line with the lack of co-expression of REM13 with REM34 and REM46, REM13 did not heterodimerize with any of those, suggesting an independent role.

## Discussion

Here, we report roles in flowering time regulation for *REM13*, *REM34* and *REM46* potentially through the modulation of cell division. Based on the phenotype of the mutants, co-expression and protein interaction data, we propose the existence of independent networks involving different sets of REM proteins that influence flowering time in Arabidopsis.

All investigated plant genomes contain dozens of *REM* genes, many located in tandem, suggesting that they arose through gene duplication events, (Ahmad et al. 2019; Romanel et al. 2009; Swaminathan et al. 2008; Verma and Bhatia 2019; Wang et al. 2012) so redundancy might be hiding the role of most of them.

For this reason, we initially focused our attention on *REM13*, a ‘solitary’ *REM* gene located in chromosome 3, as it might be less redundant than *REMs* located in tandem. We characterized two *rem13* mutant alleles, one of which showed an increase in the expression of *REM13* (*rem13-1* or *rem13_oe*), and the other a slight reduction (*rem13-2* or *rem13_kd*) and they were early and late flowering, respectively (Fig. 1).

To better understand the role of *REM13* and to investigate if it cooperates with other REMs in flowering time regulation or other traits, we performed a clustering analysis of the Arabidopsis *REM* family based on the co-expression data present in *athrna* database (Zhang et al. 2020) (Fig. 2). Most *REMs* grouped in clusters, suggesting that they might cooperate or be functionally redundant since they are co-expressed with similar sets of genes. *REM13* belonged to a small cluster containing *VDD* and *VAL*, involved in synergid identity and degeneration (Matias-Hernández et al. 2010; Mendes et al. 2016).

We also observed that although genes located closely in the genome tend to be co-expressed, on some occasions, they can be co-expressed with *REMs* at other genomic locations (i.e., *REM34* and *REM46*) (Supplementary Fig. 1), and conversely, genes like *VRN1* or *RTV1* are not co-expressed with any other *REMs* even if they are located in tandem with other *REM* genes (Supplementary Fig. 1). Overall, this suggests that the genomic position and the presence of other *REMs* close by are not entirely predictive of the potential redundancy of a particular *REM* and that the co-expression analysis proposed here might be a successful strategy to identify redundancy relationships in this highly redundant family.

Another interesting finding of the co-expression analysis is that none of the *REMs* previously described as regulators of flowering time (*VRN1*, *RTV1*, *REM16* and *TFS1*) clustered together, suggesting that they might control floral transition independently. This observation led us to wonder whether genes clustering together, such as *TFS1*, *REM34* and *REM46*, which have been linked to different biological processes, participate in more than one process throughout reproductive development, or if each gene is specialized in a specific stage but they regulate similar sets of genes. To explore this hypothesis, we checked if *REM34* and *REM46* have also a role in flowering time.

*rem34-1* and *rem46-1* mutants showed reduced flowering time in LD (Fig. 3), which correlated with the higher expression at early time points of the floral integrators *SOC1* and *AP1* compared to the wild type. This confirms that *REM34* and *REM46* have multiple roles in reproduction and suggests that this situation could be extended to other *REM* genes.

In line with its strong early flowering phenotype, *rem34-1* showed the highest upregulation of *SOC1*, *LFY* and *AP1*, which were strongly upregulated already before the transfer to LD (Fig. 4). In the case of *rem13_oe* and *rem46-1* at T0 only *SOC1* and *AP1* were upregulated, while *LFY* showed a pattern similar to the wild type.

*SOC1* and *AP1* are directly regulated by FT (Fornara et al. 2010), which was recently found to be a direct target of REM16 (Yu et al. 2020). The upregulation of *SOC1* and *AP1* in *rem34-1* and *rem46-1* might suggest these genes directly or indirectly regulate *FT*. The fact that *LFY* is not upregulated in *rem46-1* and *rem13_oe* at T0, despite being a target of *SOC1* and *AP1*, could indicate that REM13 and REM46 regulate these genes through an independent pathway.

Then, we used GO term enrichment analysis of the combined CENs of the clusters of the *REMs* under study (Fig. 5) to gather new insight on the molecular function of the clusters of interest. In the case of *REM34* and *REM46*, the analysis suggested that the genes in this cluster might be associated with cell division (Fig. 5b). Interestingly, we recently showed that REM34 modulates the expression of *Kip-related protein 6* (*KRP6*), a cyclin-dependent kinase inhibitor (CKI), (Caselli et al. 2019) and *TFS1* was also suggested to regulate cell division based on its pattern of expression in the meristem (Richter et al. 2019).

We thus analyzed whether our mutants present differences in cell division during the floral transition (Fig. 6). Although enrichment analysis did not give clues regarding the molecular role of *REM13*, we also tested if it controls cell division because *REM13* is co-expressed with only 4 *REMs* so, maybe, the role for *REM13* in cell division was not revealed due to the lower power of the enrichment analysis. Indeed, *rem13_oe*, as well as *rem34-1* and *rem46-1* showed an increased percentage of cells in the S phase at T0, suggesting that the rate of cell division in the SAM was higher than in the wild type (Fig. 6). As the increase of cell division at the SAM is one of the earliest events observed during the floral transition (Jacqmard et al. 2003; Klepikova et al. 2015) the presence of a higher percentage of cells in the S phase at T0 might explain the early flowering phenotype observed (Fig. 3). As so, our work constitutes an interesting starting point to explore the link between the higher cell division rate observed in these mutants and its effect on flowering time.

As some REM were reported to be able to homo- and/or heterodimerize (Caselli et al. 2019; Mendes et al. 2016), we checked the interaction of the REM proteins under study, as it could give further information regarding their possible cooperation. Protein interaction assays showed that REM34 heterodimerizes with REM46, suggesting that these two proteins could cooperate in the regulation of flowering time. Nevertheless, they are not completely redundant as both the mutants show an acceleration in the floral transition. The stronger phenotype of *rem34-1* and the fact it is the only mutant in which the expression of all the floral integrators analyzed (*SOC1*, *LFY* and *AP1*) is altered, suggests that *REM34* might participate in several regulatory networks. This could imply that REM34 and REM46 might modulate cell cycle progression together and that REM34 might participate in additional pathways not related to the control of the cell cycle, possibly partnering with other REMs.

We also investigated the possible interactions between TFS1 and REM34 and REM46, as they are co-expressed (Fig. 2) and all show phenotypes related to flowering time (Richter et al. 2019). TFS1 homodimerized but it did not interact with REM34 or REM46. This is coherent with their different roles in flowering time as TFS1 promotes floral transition (Richter et al. 2019) and REM34 and REM46 repress it. Finally, REM13 does not interact with itself or with any of the REMs tested in this work. This observation is not entirely surprising given the lack of co-expression with them (Fig. 2). Overall, the newly found interactions reported here, together with the interactions already reported for VDD, VAL, REM34 and REM35 suggest that dimerization could be a frequent event for the REM family.

It is remarkable that we have revealed the roles in the flowering time of *REM13/34/46* using alleles that lead to changes in their expression level (Figs. 1 and 3), rather than a loss of function. Changes in the expression level of genes have led to interesting phenotypes on several occasions during plant domestication (Manrique et al. 2019). For example, the acquisition of photoperiod-insensitivity in maize that allowed its growth at high latitudes was caused by the downregulation of *ZmCCT9* gene provoked by the insertion of a transposon in its regulatory region (Huang et al. 2017). Moreover, it was recently published that a mutation in the promoter of *OsREM20* during rice domestication led to an increase in the expression of *OsREM20* producing an increment in the number of grains per panicle (Wu et al. 2021). Similarly, our results show that the modulation of the level of expression of *REM13/34/46* leads to changes in flowering time. This could be interesting for breeding programs, as it further suggests that the modulation of the expression of *REM* genes can affect important agronomical traits.

In summary, on one hand, our strategy of relying on the lack of other REMs located in tandem with *REM13* has been successful in finding a biological role for it. On the other hand, using co-expression data as a starting point for planning functional analysis, we have uncovered roles in flowering time regulation and changes in cell division rate for *REM34* and *REM46*. Our attempt of using co-expression data as a starting point for functional analysis of *REMs* has been more successful than previous ones (Mantegazza et al. 2014), probably due to the increase in the amount and quality of transcriptomic data available. Moreover, it has revealed interesting features regarding the redundancy patterns of REM genes that open the door to better-focused studies of the *REM* family where the combination of co-expression and interaction data might help to uncover more biological roles for these cryptic transcription factors.

## Supplementary Information

Below is the link to the electronic supplementary material.

Supplementary material 1 (DOCX 2768.9 kb)

Supplementary material 2 (XLSX 12.4 kb)

Supplementary material 3 (XLSX 10.3 kb)

Supplementary material 4 (XLSX 19.1 kb)

Supplementary material 5 (XLSX 19.9 kb)
