# Supplementary material for: Assessing the role of REM13, REM34 and REM46 during the transition to the reproductive phase in Arabidopsis thaliana
Source: Plant Mol Biol. 2023 May 12;112(3):179–93. doi: 10.1007/s11103-023-01357-1 (PMC10267284; doi:10.1007/s11103-023-01357-1)
Supplement: Supplementary file 1 — Supplementary material 1 (DOCX 2768.9 kb) [file 11103_2023_1357_MOESM1_ESM.docx]

Assessing the role of *REM13*, *REM34* and *REM46* during the transition to the reproductive phase in *Arabidopsis thaliana*

Silvia Manrique ^1,4^, Francesca Caselli ^1,4^, Luis Matías-Hernández^1,3^, Robert G Franks^2^, Lucia Colombo^1^ and Veronica Gregis ^1,*^

^1^ Dipartimento di Bioscienze, Università degli Studi di Milano, Via Giovanni Celoria 26, 20133, Milan, Italy

^2^ North Carolina State University, Department of Plant and Microbial Biology, Raleigh, NC, 27606, USA

^3^ Tricopharming, C/Pallars 99, 08018, Barcelona

^4^  These authors contributed equally to the work

^*^ Correspondence: [veronica.gregis@unimi.it](mailto:veronica.gregis@unimi.it), Orcid ID: https://orcid.org/0000-0003-1876-9849

**Supplementary Information**

**Supplementary Table 1** List of primers employed in this work

**Supplementary Table 2.** Non-redundant list of *REM* genes detected by, Swamithaman et al., 2008; Romanel et al., 2009 and Wang et al., 2012

**Supplementary Table 3** CEN of each *REM* gene, retrieved from the *athrna* database

**Supplementary Table 4** Analysis of Biological Process GO terms associated with the genes belonging to the CENs of the clusters containing *REM13* and *TFS1*


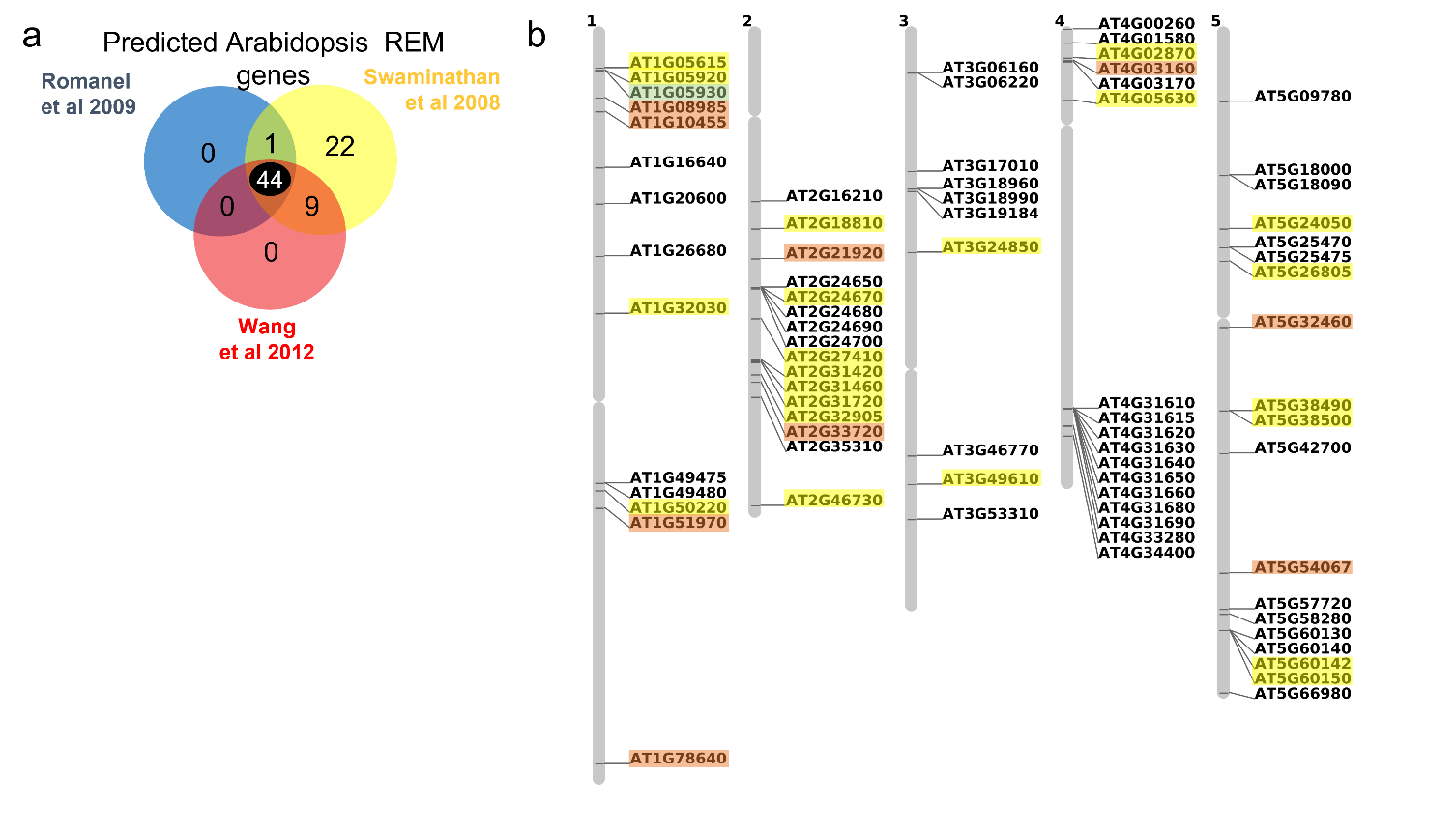
**Supplementary Fig 1** Predicted *REM* genes from Arabidopsis. (a) Venn diagram showing the overlap of *REM* gene predictions obtained by Swaminathan et al (2008), Romanel et al (2009) and Wang et al (2012). (b) Position in the chromosomes of all predicted *REM* genes of *A. thaliana*. Genes in black represent the 44 REM genes predicted by all authors. Genes highlighted in yellow represent genes predicted only by Swaminathan et al (2008), genes highlighted in orange are the ones predicted by both Swaminathan and Wang Works, and genes highlighted in green represent genes predicted by both Romanel and Swaminathan studies, following the same color pattern depicted in (a)

**
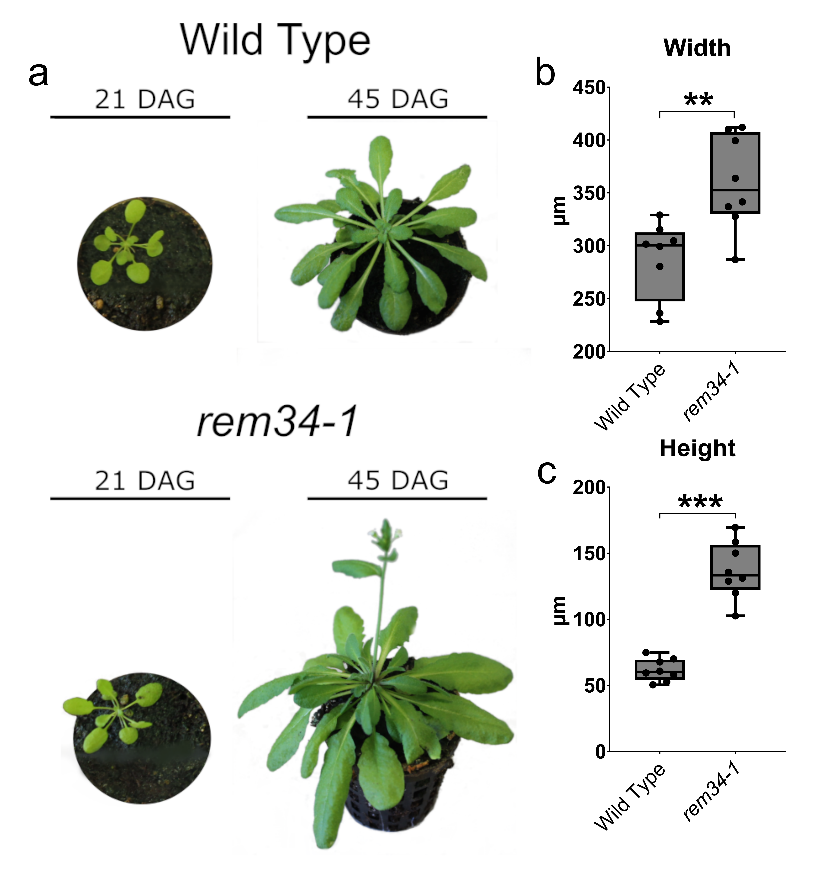
**
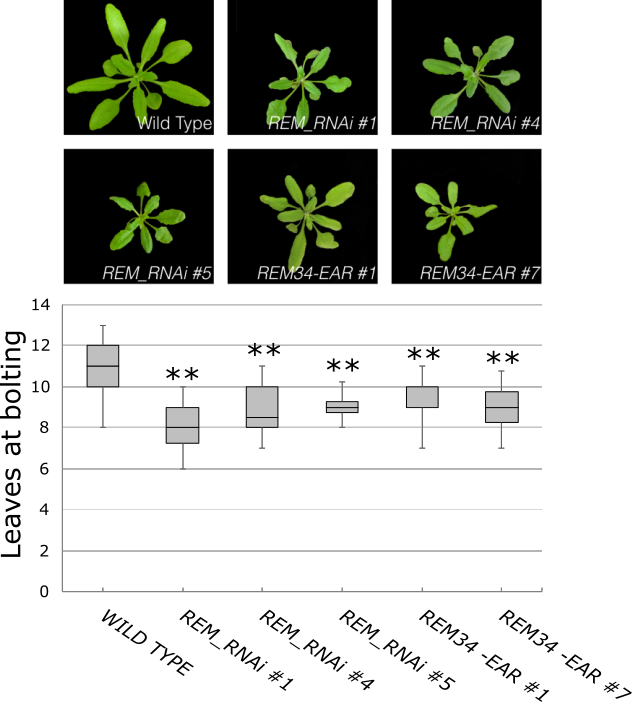
**Supplementary Fig 2** Flowering time of REM_RNAi and 35S:REM34-EAR. (a) Wild-type, REM_RNAi and 35S:REM34-EAR rosette at bolting. (b) Flowering time of wild-type, REM_RNAi lines #1 #4 and #5 and of 35S:REM34-EAR #1 and #7 lines. Significance was calculated using a t-test coupled with Bonferroni correction for multiple testing ** p-value < 0.01

**Supplementary Fig 3** *rem34-1* characterization in SD conditions. (a) wild type and *rem34-1* plants in SD non-inductive conditions 21 and 45 Days After Germination (DAG), while at 45 DAG wild type plants are still in the vegetative phase *rem34-1* mutants show an early flowering phenotype. (b) (c) width and height of the SAM of wild type and *rem34-1* plants at 21 DAG under SD non-inductive conditions. *rem34-1* meristems showed a dramatic enlargement, consistent with their early flowering time phenotype observed in SD. Width was measured as the distance between the two leaves primordia and height was measured from the top of the meristem to the width line. Statistical significance was measured with a t-test (** p<0.01, ***<0.001).

**
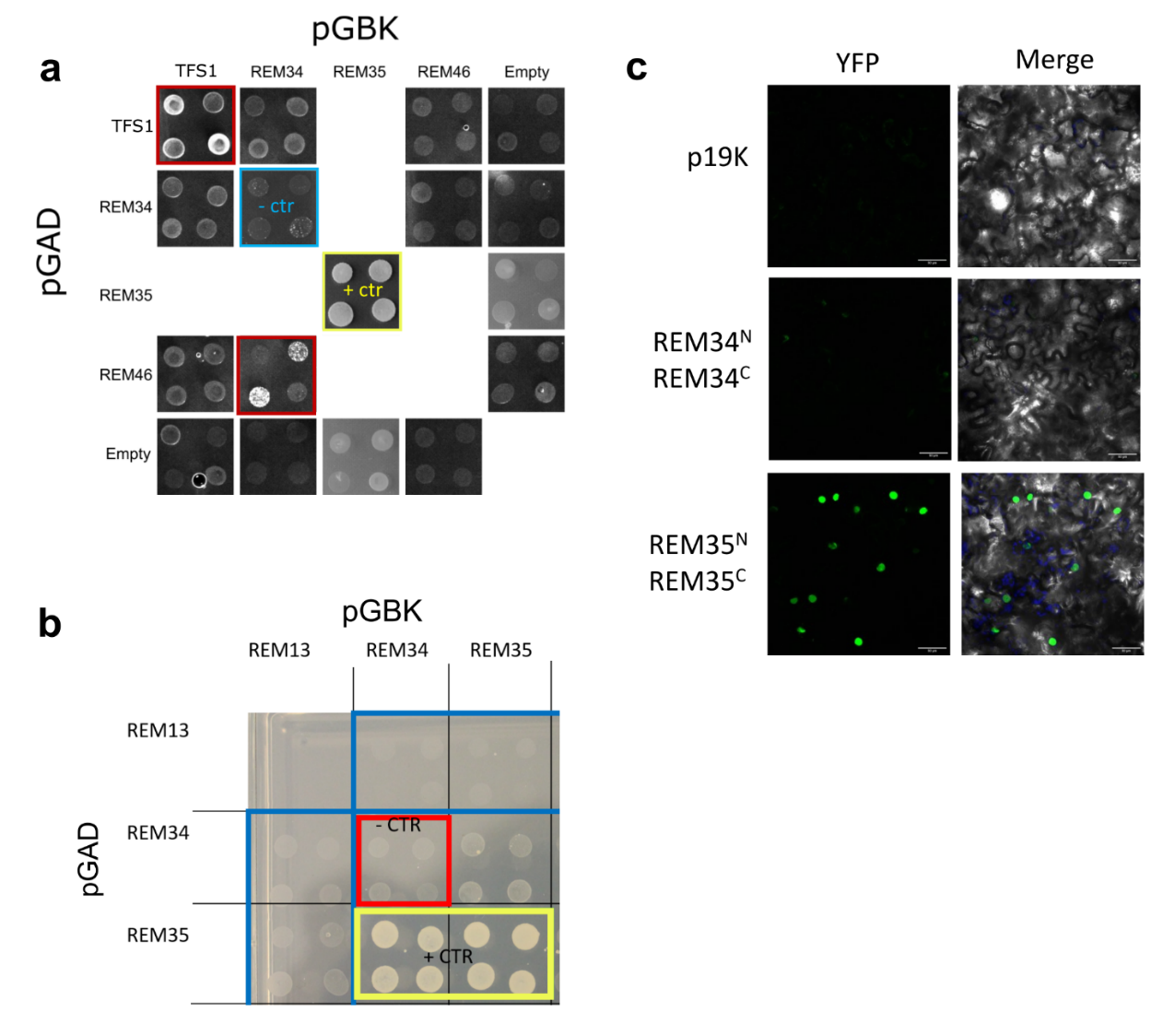
**


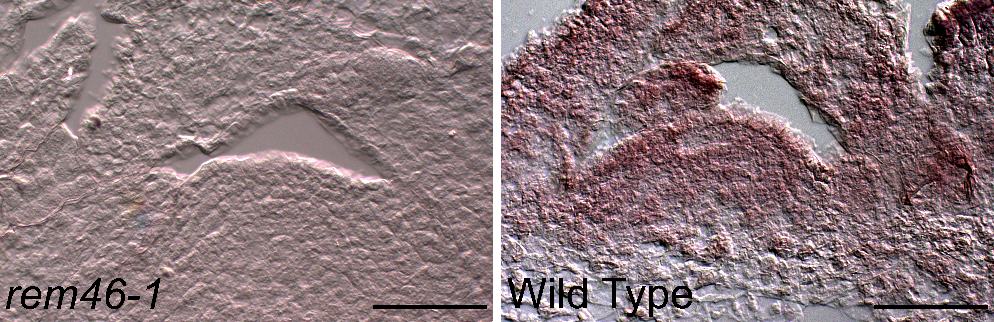
**Supplementary Fig 4** Protein-protein interactions negative controls. (a) Complete plate showing the interactions between the TFS1 cluster and all the controls employed for the experiment. Red squares indicate newly found positive interactions, blue square shows the REM34-REM34 interaction, used as negative control and yellow square indicates the REM35-REM35 positive control. (b) Complete plate showing the interactions between REM13 and REM34 and REM35. No new interactions were found (blue squares), red square indicates REM34-REM34 interaction, used as negative control and yellow squares indicate the REM34-REM35 and REM35-REM35 positive controls. (c) BiFC controls showing that no fluorescence was detected in the p19K-only infiltrated tobacco leaves. REM34C-REM34N was used as a negative control and REM35N-REM35C was used as positive control. Scale bar= 50 μM

**Supplementary Fig 5** Specificity of the *REM46* probe was assessed by comparing the signal detected in the wild type SAM to the one observable in the *rem46-1* mutant. Compared to the wild type tissue, where the colorimetric signal is clearly detectable in the meristematic tissue, no colorimetric reaction was visible in all the *rem46-1* analyzed tissue sections, suggesting that the probe is highly specific for the *REM46* mRNA. Scale bar= 100 μM

**References**

Romanel, E., Schrago, C. G., Couñago, R. M., Russo, C. A. M., & Alves-Ferreira, M. (2009). Evolution of the B3 DNA binding superfamily: New insights into REM family gene diversification. *PLoS ONE*, *4*(6). https://doi.org/10.1371/journal.pone.0005791

Swaminathan, K., Peterson, K., & Jack, T. (2008). The plant B3 superfamily. *Trends in Plant Science*, *13*(12), 647–655. https://doi.org/10.1016/j.tplants.2008.09.006

Wang, Y., Deng, D., Zhang, R., Wang, S., Bian, Y., & Yin, Z. (2012). Systematic analysis of plant-specific B3 domain-containing proteins based on the genome resources of 11 sequenced species. *Molecular Biology Reports*, *39*(5), 6267–6282. https://doi.org/10.1007/S11033-012-1448-8
